# Optimal peripheral nerve stimulation intensity for paired associative stimulation with high-frequency peripheral component in healthy subjects

**DOI:** 10.1038/s41598-022-16811-1

**Published:** 2022-07-21

**Authors:** Markus Pohjonen, Anna-Lena Nyman, Erika Kirveskari, Jari Arokoski, Anastasia Shulga

**Affiliations:** 1 Department of Physical and Rehabilitation Medicine, Helsinki University Hospital and University of Helsinki, PO Box 349, 00029 HUS Helsinki, Finland; 2 BioMag Laboratory, HUS Diagnostic Center, University of Helsinki and Helsinki University Hospital, Helsinki, Finland; 3 Clinical Neurophysiology, Clinical Neurosciences, HUS Diagnostic Center, Helsinki University Hospital and University of Helsinki, Helsinki, Finland; 4 Faculty of Medicine, University of Helsinki, Helsinki, Finland; 5 Validia Rehabilitation Center, Helsinki, Finland

**Keywords:** Spinal cord diseases, Synaptic plasticity

## Abstract

Paired associative stimulation (PAS) with high-frequency peripheral nerve stimulation (PNS), called “high-PAS”, induces motor-evoked potential (MEP) potentiation in healthy subjects and improves muscle activity and independence in incomplete spinal cord injury patients. Data on optimal PNS intensity in PAS are scarce. In a high-PAS protocol, PNS intensity is defined as “minimal intensity required to produce F-responses”. We sought to further refine this definition and to investigate how PNS intensity affects PAS outcome. Two experiments were performed on 10 healthy subjects where MEP amplitude change was measured 0, 30, and 60 min after PAS. In the first experiment, the intensity required to achieve 7/10 persistence of F-responses was used to define PNS intensity level. In the second experiment, we used the intensity required to achieve 1/10 persistence (“baseline”). In addition, we applied this intensity at + 25%, − 25%, and − 50% levels. In the first experiment, PAS did not produce significant MEP potentiation. In the second experiment, PAS produced statistically significant MEP potentiation, with PNS intensity of “baseline” and “baseline − 25%” levels but not at + 25% or − 50% levels. In conclusion, for PAS utilizing high-frequency PNS, the intensity required to achieve 1/10 F-response persistence or the intensity 25% lower produces significant MEP potentiation in healthy subjects.

## Introduction

Paired associative stimulation (PAS) is a combination of transcranial magnetic stimulation (TMS) and peripheral nerve stimulation (PNS)^1^. In spinal PAS, the stimulation is induced synchronously at the motor cortex and corresponding peripheral nerve such that ascending and descending volleys collide at corticomotoneuronal synapses at the spinal level^2^. PAS promotes long-term potentiation (LTP)-like plasticity in stimulated corticomotoneuronal synapses^1,3,4^. Different variations of PAS are under investigation as tools to improve various neurological deficits^4^.

While conventional PAS protocols are strictly dependent on the timing between TMS and PNS (interstimulus interval; ISI) and numerous other factors^4^, we have developed our own PAS modification that consists of high-intensity TMS and high-frequency PNS (“high-PAS”)^5^. High-PAS is resilient to variability in ISIs and small inaccuracies in mapping, making it feasible for clinical practice^6,7^. High-PAS applied as a long-term treatment can enhance motor output in weak muscles and return some movement to paralyzed muscles after incomplete spinal cord injury (SCI), most plausibly through strengthening synaptic contacts between upper and lower motor neurons at the spinal level^5,8–13^.

Although most SCI patients tolerate PAS well^5^, it may be necessary to lower PNS intensity at least in the beginning when the patient is getting used to stimulation. On the other hand, some patients may even ask to increase intensities over the pre-defined values, since they intuitively think that this could improve the result. Data on optimal PNS intensity and dose–response studies in PAS research are minimal^4^. Previously, various levels of PNS intensity in PAS have been used, e.g. the motor threshold level^14^ or twice the sensory threshold level^15^.

We have used minimum intensity eliciting F-waves in high-PAS^16^. Since F-wave is a response of orthodromic activation of antidromically activated motor neurons, this ensures that stimulation reaches the lower motoneurons of the spinal cord^6^. We have not investigated optimal intensity of PNS in our protocol. Here, we sought to determine if lowering or increasing the PNS intensity would affect the extent of motor evoked potential (MEP) potentiation by PAS in healthy subjects and to define the optimal PNS intensity for high-PAS more precisely in relation to F-response persistence.

## Materials and methods

### Subjects

The study was approved by the medical ethical committee of the Helsinki University Hospital. All experiments were performed in accordance with relevant guidelines and regulations. All subjects provided signed informed consent. Thirteen healthy subjects without contraindications to TMS were recruited for two experiments (Experiment 1: 10 subjects, age range 18–55 years, 7 females, resting motor threshold [RMT] 54–95%, RMT mean 70%; Experiment 2: 10 subjects, age range 18–55 years, 8 females, RMT 54–90%, RMT mean 66%). In Experiment 2, 7 subjects were the same as in Experiment 1. Subjects were advised to avoid caffeine intake (coffee or energy drinks) for 6 h prior to the measurements^17^ and to not engage in intensive physical activity for 1 day prior to the measurements. The measurements were performed at the same time of day (± 2 h maximum). There was at least a 1-week period between each stimulation visit to prevent possible carryover effect of the previous stimulation.

### F-response measurement and peripheral nerve stimulation (PNS)

A Dantec Keypoint device (Natus Medical Inc., Pleasanton, CA) was used for PNS and F-response measurement. We electrically stimulated the tibial nerve using two surface electrodes (Neuroline 720, AMBU A/S, Ballerup, Denmark) placed on the medial surface of the right ankle behind the medial malleolus. For the F-response recording, the active electrode was placed on the bulk of the abductor hallucis (AH) muscle and the reference electrode on the median bony surface of the big toe.

First, the F-waves were detected using 0.2-ms pulses at supramaximal intensity. Then, using 1-ms pulses (same pulse width as in PNS component of PAS), the minimal intensity eliciting comparable F-waves was identified. The shortest latency out of 10 F-responses evoked with a single 0.2-ms pulse at suprathreshold intensity was used for calculation of ISI between PNS and TMS^6^. For PNS, 100-Hz stimulation was used, as this was the most effective frequency in high-PAS^7,18^.

In Experiment 1, PNS intensity was defined as an intensity eliciting minimum 7 F-waves out of 10 when 1-ms pulses were administered (7/10 persistence). In Experiment 2, PNS intensity was determined as an intensity eliciting at least 1 F-wave out of 10 pulses administered (1/10 persistence). This level was determined by administering 1-ms pulses first in ascending 0.5-mA steps until F-responses were detected and subsequently in descending 0.5-mA steps to find the lowest threshold for 1/10 persistence. The 1-ms pulses were given at variable intervals of approximately 2 s. In Experiment 2, subjects’ F-response stimulation intensities were determined each time before PAS. Motor thresholds (minimal intensity for observed movement of ankle or toes) were identified using 1-ms pulses. Lidocaine/prilocaine 2.5% creme (EMLA) was applied to subjects’ stimulated area in both experiments to prevent unpleasant sensations during PNS. EMLA penetrates 3–5 mm into the skin and thus does not affect the conductivity of the tibial nerve^19^.

**Figure 1:**
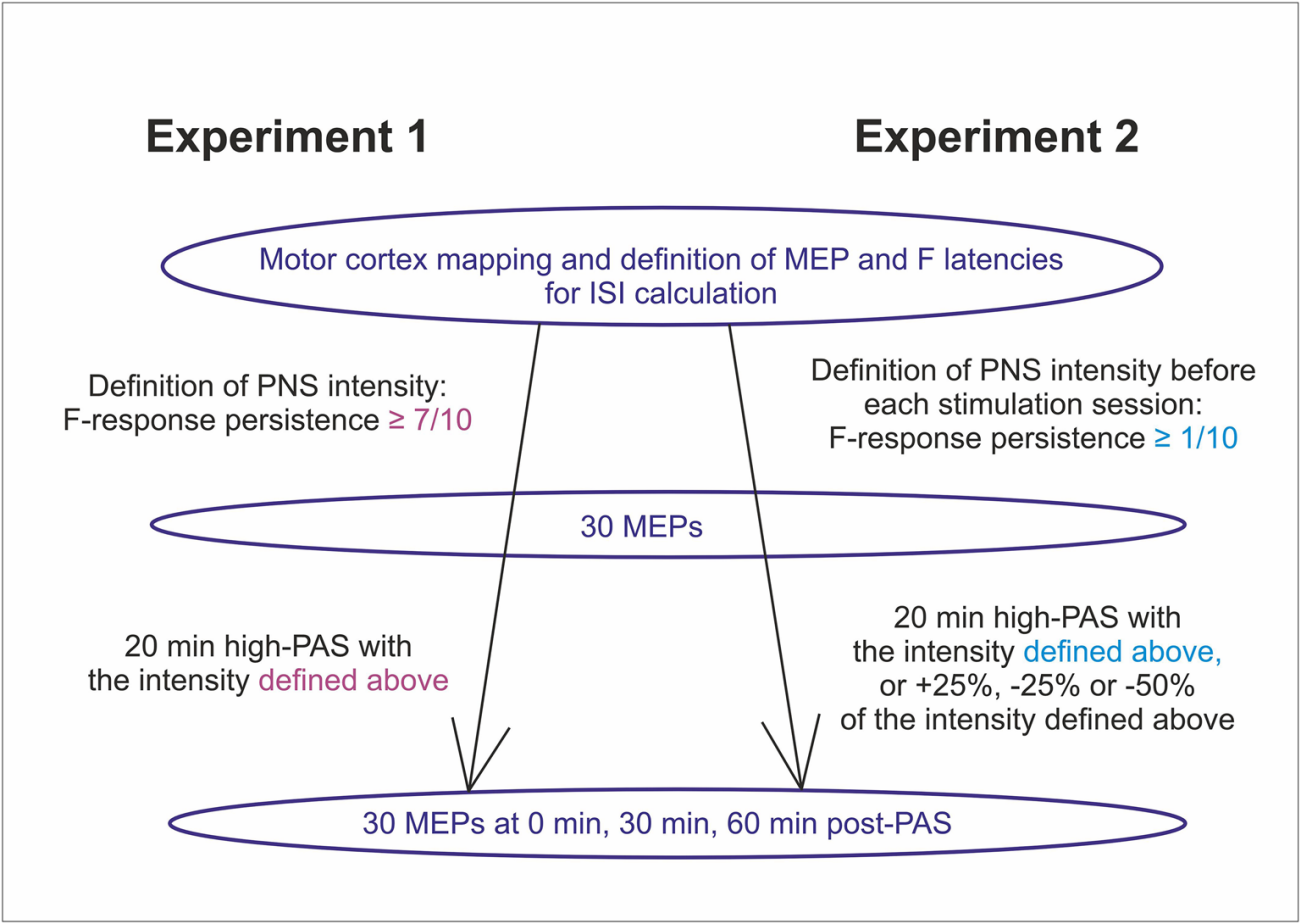
Experimental setup. *AH* abductor hallucis muscle, *MEP* motor evoked potential, *ISI* interstimulus interval, *RMT* resting motor threshold.

See Fig. 1 for experimental setup.

### Transcranial magnetic stimulation (TMS)

TMS was administered with a navigated figure-8 coil with eXimia magnetic stimulator (Nexstim Ltd., Helsinki, Finland). Suprathreshold stimuli were applied throughout the representation area of the leg on primary motor cortex (M1) for mapping. After finding the stimulation area corresponding to plantar flexion movement and measurable MEPs, the specific hotspot in M1 was determined where TMS produced consistent and the largest MEPs. RMT of the hotspot was determined as the lowest TMS intensity that produced MEPs of at least 50 µV in a minimum of 5/10 attempts.

### Motor evoked potentials (MEPs)

After determining AH hotspot in M1, MEPs were elicited by TMS to this hotspot at 120% RMT. Thirty MEPs were recorded with a 3.3-s interval. The EMG signal was visually screened in a 200-ms time window before TMS pulse for possible increase of EMG activity exceeding baseline. MEP recordings that had excessive EMG activity before the TMS pulse were omitted to exclude effects of muscle preactivation. MEP potentiation was defined as the percent ratio of an average of post-PAS to pre-PAS MEPs. Throughout the manuscript, all results are presented as % post-PAS of pre-PAS. MEPs were recorded before the high-PAS session, immediately post-PAS, 30 min, and 60 min post-PAS.

### PAS

PAS was administered as previously^5^ except for PNS intensity. A single-pulse TMS at 100% of stimulator output (SO) and PNS in 100-Hz trains of 6 pulses (pulse width 1 ms) were paired every 5 s (0.2 Hz). ISI was calculated for each subject as previously with the formula ISI = F-response latency − MEP latency)^5^ to provide synchronous arrivals of the first descending activity induced by TMS and first ascending activity induced by PNS at the spinal-cord level. TMS and PNS were triggered with Presentation software (Neurobehavioral System Inc., Albany, NY). One PAS session consisted of 240 paired pulses (20 min in total).

**Figure 2:**
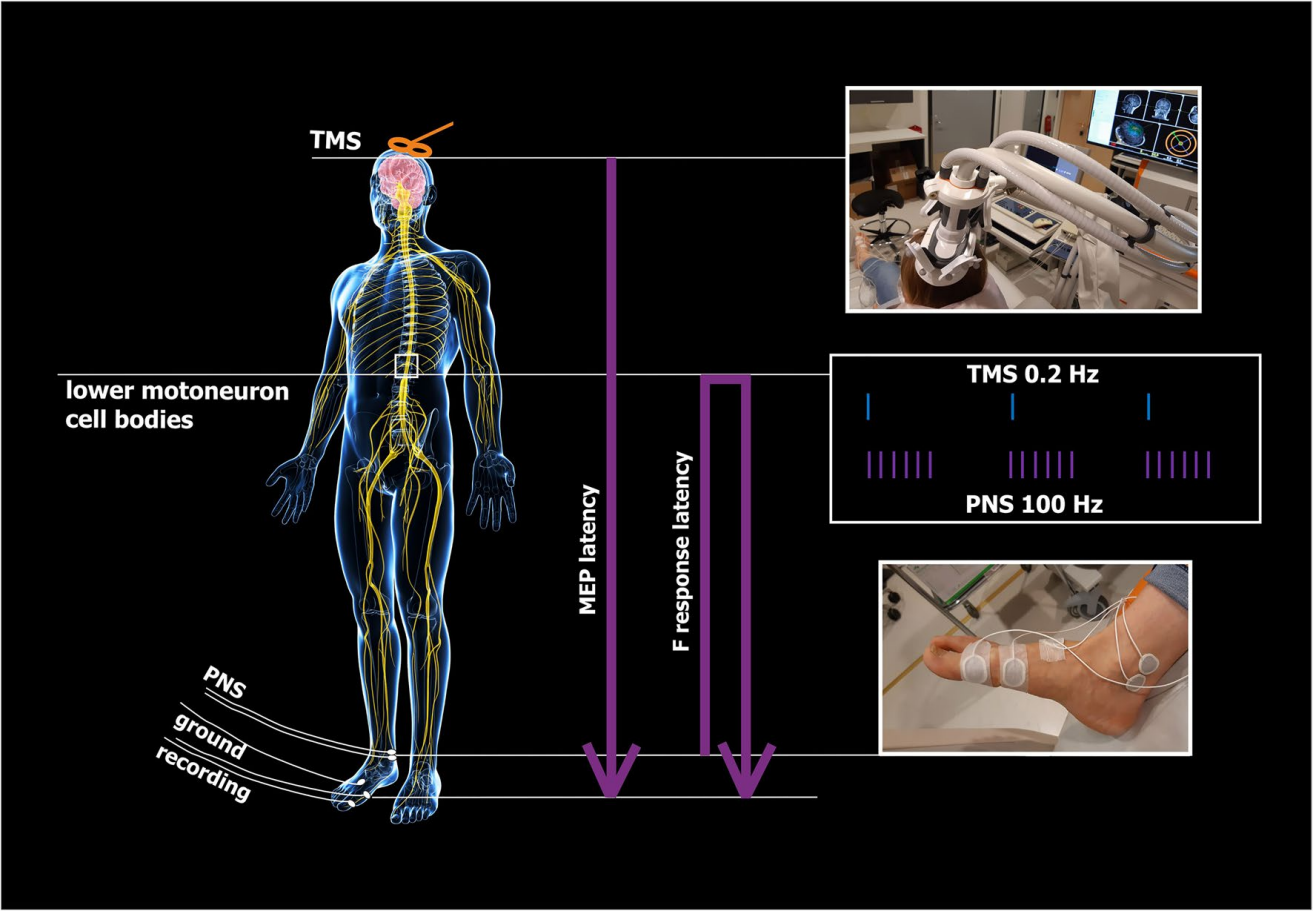
Schematic representation of high-PAS setup. Upper motor neurons are activated by transcranial magnetic stimulation (TMS, represented as an orange-coloured coil, see also photograph of the coil and navigation system on the right). Lower motor neurons are activated by peripheral nerve stimulation (PNS, here stimulation electrodes are located behind the medial malleolus to activate tibial nerve, see also photograph on the right). Recording electrodes are placed at the abductor hallucis (AH) muscle belly and are used for F-response and motor-evoked potential (MEP) latency recordings. F-wave latency and motor evoked potential (MEP) latency are measured before the PAS session to calculate the interval between TMS and PNS with the formula [interstimulus interval (ISI) = F_latency_ − MEP_latency_] as described before in Refs.^5,6^. During PAS, TMS pulses arrive every 5th second to corticomotoneuronal synapses, coinciding with the first pulse of the 100 Hz-PNS train. See Refs.^5,6,13^ for more figures and a detailed description of the high-PAS setup.

See Fig. 2 for a schematic representation of PAS protocol.

### Statistics

Data were analysed with IBM SPSS Statistics software. All data were non-parametric according to Shapiro–Wilk test. We used Friedman test for multiple comparisons (MEP potentiation across all time points, i.e. pre-PAS and 0 min, 30 min, and 60 min post-PAS) and Wilcoxon signed-ranked test for pairwise comparisons (each time point *vs* pre-PAS for those conditions where Friedman test produced a p-value of < 0.05). The data in the manuscript is presented as mean ± standard error.

### Ethical approval

Helsinki University Hospital Ethical committee.

## Results

First, we hypothesized that aiming at 7/10 persistence in F-response measurement and using this as baseline PNS intensity would produce stable PAS results. To test this, in Experiment 1 we selected the minimal PNS intensity level required to produce 7/10 F-response persistence and performed PAS in 10 subjects to assess the stability of MEP potentiation under these settings. F-response measurement and PAS were performed on different days. In contrast to our previous results^7,18^, this protocol did not produce significant MEP potentiation (p = 0.28 by Friedman test); post-PAS normalized to pre-PAS was 131% ± 15% at 0 min, 105 ± 14% at 30 min, and 112 ± 16% at 60 min (n = 10 subjects, here and elsewhere in the manuscript the data is presented as mean ± standard error). See Supplementary Table A1 for raw data.

Next, we hypothesized that since the PNS level required to produce 7/10 persistence was higher than the level that we used in our previous studies (just sufficient to elicit F-responses), this higher level might not be beneficial, contrary to our initial assumption. We measured F-responses from 10 subjects to determine the relationship between the level required to produce at least one F-response (1/10 persistence) and the level required for 7/10 persistence. We measured each subject four times on different days. The level required to produce 7/10 persistence was on average 24 ± 2% higher than the 1/10 level (n = 40 measurements in 10 subjects). There was some within-subject day-to-day variability in threshold levels; the result of second to fourth measurement normalized to first measurement was on average 99 ± 20% (n = 40 measurements in 10 subjects). There was no systematic change of thresholds upon multiple measurements (p = 0.6 by Friedman test). The 1/10 level was on average 120 ± 4% of motor threshold. See Supplementary Table A2 for raw data.

**Figure 3:**
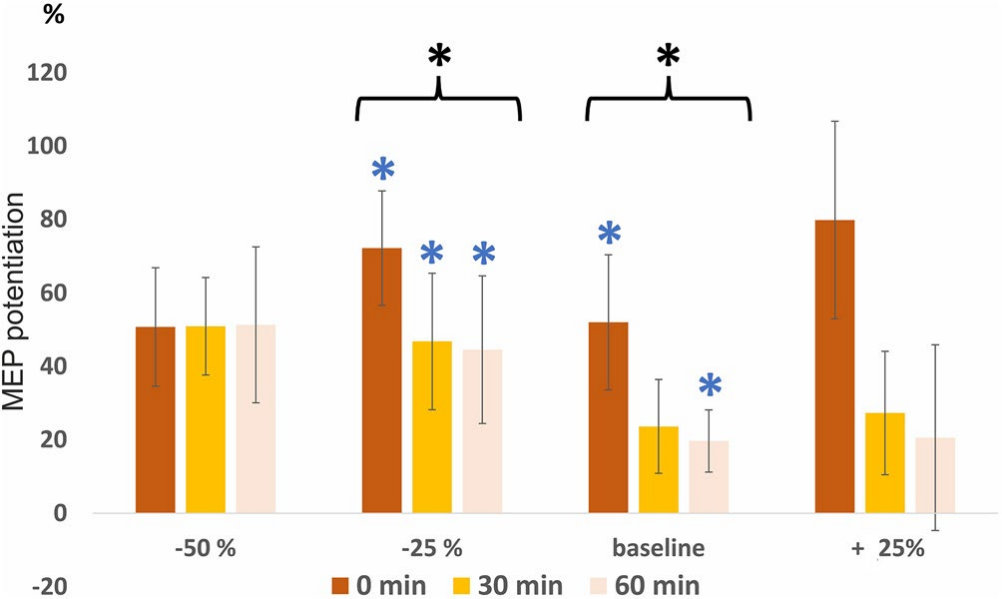
Results of Experiment 2. The data is presented as mean ± standard error. Motor evoked potential (MEP) potentiation is visualized as % post paired associative stimulation (PAS) of pre-PAS minus 100% (everywhere else in the manuscript, all results are presented as % post-PAS of pre-PAS without subtracting 100%). Baseline intensity means minimal intensity required to produce 1/10 F-response persistence. Considering all time points as a group, PAS produced statistically significant MEP potentiation (black stars) with peripheral nerve stimulation (PNS) intensity of “baseline” and “baseline − 25%” levels (p = 0.033 and p = 0.018 by Friedman test, respectively), but not with PNS intensity of + 25% and − 50% levels. Within the “baseline” group (blue stars), there was a significant MEP potentiation at 0 min and 60 min (p = 0.009 and 0.041 by Wilcoxon signed-rank test, respectively). Within − 25 level results (blue stars), there was significant MEP potentiation at all time points (p = 0.007, 0.037, 0.041, at 0, 30, and 60 min, respectively, by Wilcoxon signed-rank test).

In Experiment 2, we applied PAS at the 1/10 persistence level (“baseline”) and at 25% higher (“+ 25”) and 25% (“− 25”) and 50% (“− 50”) lower levels (Fig. 3, see Supplementary Table A3 for raw data). PAS induced significant MEP potentiation at baseline and − 25 levels (p = 0.033 and p = 0.018 by Friedman test, respectively) but not at + 25 level (p = 0.19 with Friedman test) or − 50 level (p = 0.052 with Friedman test). Within baseline results, there was a significant MEP potentiation at 0 min and 60 min (p = 0.009 and 0.041 by Wilcoxon signed-rank test, respectively). Within − 25 level results, there was significant MEP potentiation at all time points (p = 0.007, 0.037, 0.041 at 0, 30, and 60 min, respectively, by Wilcoxon signed-rank test). PAS with − 50% intensity PNS pulses also produced a trend in MEP potentiation at all time points but failed to reach statistical significance. Taking all time points into analysis, the extent of MEP potentiation at − 25 level was not significantly higher than that at baseline level (p = 0.082 by Wilcoxon signed-rank test). The result of + 25 level is consistent with the result of the Experiment 1, where the intensity was also approximately 25% higher than the level of 1/10 (see above).

## Discussion

We found that the PNS level eliciting 1/10 F wave persistence and the level 25% below it were the most effective stimulation levels for MEP potentiation. This demonstrates that, perhaps counterintuitively, applying higher PNS intensities in high-PAS “just in case” is not beneficial in healthy subjects. Clinical dose–response studies performed in SCI patients should be conducted to confirm whether the same applies under conditions where the connectivity between upper and lower motor neurons is compromised. Based on our results, it is plausible that lowering PNS stimulation intensity (− 25%) will not diminish clinical effectiveness of high-PAS, although this still needs to be confirmed in clinical studies. Since F-response thresholds are determined with single-pulse stimulations, and PNS in high-PAS is administered as 6-pulse trains, it is highly probable that pulses sum up and still reach the spinal cord level at − 25% intensity, exciting lower motor neuron cell bodies. Lower intensities within − 25% range could plausibly be utilized in the beginning of stimulation series if the patient does not tolerate target stimulation intensities.

Our result is fully consistent with vagus nerve stimulation (VNS) studies that have shown that excessively high and excessively low stimulation intensities are less effective or even fail to produce plasticity^20–22^. As proposed in these studies, one explanation of poorer effectiveness at high intensities is the overactivation of neuromodulatory systems, which leads to desensitization of neural pathways. It is plausible that overactivation of motor neuron pool by excessively high intensities leads to compensatory hyperpolarization and similarly hampers PAS effect. Psychological stress induced by higher stimulation intensities may also have affected the result^23^. This result is also consistent with our work where we have shown that increasing PNS frequency from 100 to 400 Hz, or PAS frequency from 0.2 to 0.4 Hz is not beneficial^18^; excessively intensive stimulation may oversaturate the machinery of e.g. protein synthesis required to produce long-term plastic changes. Notably, here especially the long-term MEP potentiation at 60 min was hampered by excessively high intensity (Fig. 3).

In Experiment 2, we remeasured F-response parameters before each session. In clinical work, It is not feasible to remeasure F-responses before each stimulation, and we obtained good clinical results^5^ without remeasuring F-responses each time; when repeated long-term for several weeks, this protocol is tolerable to daily variability in thresholds. We have shown before that after 4 weeks of stimulation in SCI patients, there was no significant change in F-response persistence (11). It is noteworthy that here the + 25% level also resulted in a trend in potentiation, although due to higher variability no statistical significance was achieved. There is plausibly day-to-day and within-session variation at the single-patient level and when administered long-term, at least part of the stimulation sessions fall within the optimal range, provided the basic PNS level is defined appropriately (not excessively high or excessively low).

High-PAS technique used here has been documented to have therapeutic effects in patients with incomplete SCI with wide range of injury severity, age, and time since injury^5,8–13,24^. Upper- or lower-limb high-PAS in tetraplegic and paraplegic, traumatic, and neurological SCI patients has led to increases in manual motor scores (MMT) of upper and lower limbs, functional hand tests, walking tests, and measures of functional independence^5^. Further clinical trials are ongoing. The core idea of high-PAS settings is that multiple antidromic activations triggered by high-frequency PNS trains and multiple orthodromic volleys triggered by 100% SO TMS pulses lead to multiple interactions at the level of corcticomotoneuronal synapses^7,25^. Since interactions leading to long-term potentiation (LTP)-like effects overcome their long-term depression (LTD)—like counterparts, the net result of multiple collisions at the level of the spinal cord is an LTP-like effect^26^. High-PAS is thought to strengthen corticomotoneuronal synapses primarily at the spinal level due to the use of the [F latency − MEP latency] formula^6^, possibly having also cortical level effects^5,27^. At the spinal level, high-PAS might both reroute interneurons^28^ and directly strengthen synaptic connections between upper and lower and motor neurons, which leads to the net effect of improved corticospinal conduction^5^.

Previous research of high-PAS in healthy subjects has optimized PNS and PAS frequencies^6,7,18,25^, now this work adding to understanding of optimal PAS intensities. Although direct confirmation of the clinical relevance of healthy subject data requires additional trials in patients, it is plausible that the data is directly translatable to patients because both in patients and in healthy subjects we use individualized parameters: e.g., an SCI patient with severe injury would have higher F-response threshold and thus receive higher stimulation intensity. Existing high-PAS data has already shown that parameters that effectively potentiate MEPs in healthy subjects also benefit patients; future trials could be including simultaneously both healthy subjects and SCI patients in the same high-PAS studies to align the results even better.

## Conclusions

PNS intensity affects PAS outcome. Excessively high or excessively low levels of PNS intensity are not beneficial. However, these results were obtained in healthy subjects only and require confirmation as dose–response studies in patients with SCI. Supporting our previous data, PAS utilizing PNS intensity required to achieve 1/10 F-response persistence (“just above the threshold to produce F-responses”) produced significant MEP potentiation; this intensity can be diminished by 25% if required. The results presented here are one step further towards developing more stable and reliable PAS protocols in the clinical setting and understanding their action.

## Supplementary Information

Supplementary Tables.
