# Supplementary material for: Optimal peripheral nerve stimulation intensity for paired associative stimulation with high-frequency peripheral component in healthy subjects
Source: Sci Rep. 2022 Jul 21;12:12466. doi: 10.1038/s41598-022-16811-1 (PMC9304330; doi:10.1038/s41598-022-16811-1)
Supplement: Supplementary file 1 — Supplementary Tables. [file 41598_2022_16811_MOESM1_ESM.docx]

**Supplementary Table legends**

**Supplementary Table A.1.** Raw data of Experiment 1.

The data include average amplitudes of 30 motor evoked potentials (MEPs) measured before PAS for 10 subjects (see Methods) and respective post-paired associative stimulation (PAS) values in % normalized to pre-PAS. STD – standard deviation, STE – standard error.

**Supplementary Table A.2.** Raw data of F-response measurements.

10 subjects underwent 4 measurements each. For each subject, we detected the intensity required for 1/10 F-response persistence and for 7/10 F-response persistence. We also measured the motor thresholds (see Methods).

**Supplementary Table A.3.** Raw data of Experiment 2.

The data include average amplitudes of 30 motor evoked potentials (MEPs) measured before paired associative stimulation (PAS) for 10 subjects (see Methods) and respective post-PAS values in % normalized to pre-PAS. STD – standard deviation, STE – standard error.

SUPPLEMENTARY TABLE A.1

| **Experiment 1** | |  |  |  |
| --- | --- | --- | --- | --- |
|  | MEP Amplitudes | % post-PAS to pre-PAS | | |
|  |  | **0 min** | **30 min** | **60 min** |
| **Subject** | **pre-PAS baseline (µV)** | **%** | **%** | **%** |
| 1 | 333 | 165 | 147 | 98 |
| 2 | 203 | 195 | 173 | 165 |
| 3 | 114 | 184 | 168 | 119 |
| 4 | 624 | 136 | 98 | 234 |
| 5 | 116 | 150 | 91 | 76 |
| 6 | 382 | 77 | 53 | 110 |
| 7 | 145 | 71 | 117 | 99 |
| 8 | 377 | 69 | 72 | 69 |
| 9 | 206 | 150 | 83 | 90 |
| 10 | 578 | 114 | 48 | 60 |
| Mean |  | 131 | 105 | 112 |
| **Mean – 100%** |  | **31** | **5** | **12** |
| STD |  | 46 | 45 | 52 |
| **STE** |  | **15** | **14** | **16** |

SUPPLEMENTARY TABLE A.2

|  |  |  |  |  |
| --- | --- | --- | --- | --- |
| **Subject** | **Measurement** | **Intensity required to reach 1/10 F-response persistence (mA)** | **Intensity required to reach 7/10 F-response persistence (mA)** | **Motor threshold (toe or ankle movement observed) (mA)** |
| **1** | 1 | 8 | 11 | 8.5 |
|  | 2 | 11.5 | 13.5 | 9 |
|  | 3 | 10.5 | 11.5 | 9.5 |
|  | 4 | 8.5 | 10.6 | 8.5 |
| **2** | 1 | 5.5 | 8.5 | 6.5 |
|  | 2 | 5 | 6.3 | 5 |
|  | 3 | 6.5 | 8.1 | 5 |
|  | 4 | 5 | 6.3 | 5.5 |
| **3** | 1 | 8.5 | 12.5 | 7.5 |
|  | 2 | 11.5 | 14 | 6 |
|  | 3 | 7.5 | 11 | 6.5 |
|  | 4 | 11.5 | 16 | 7 |
| **4** | 1 | 8 | 8.5 | 8.5 |
|  | 2 | 8.5 | 9 | 9 |
|  | 3 | 5.5 | 5.5 | 5.5 |
|  | 4 | 6 | 6 | 6.5 |
| **5** | 1 | 4 | 5 | 3.5 |
|  | 2 | 3.5 | 4.5 | 3.5 |
|  | 3 | 3.5 | 4.4 | 3 |
|  | 4 | 5 | 6.5 | 4.5 |
| **6** | 1 | 4.5 | 5.6 | 3 |
|  | 2 | 5.5 | 6 | 3 |
|  | 3 | 4.5 | 6 | 3 |
|  | 4 | 4.5 | 5.5 | 3 |
| **7** | 1 | 5.5 | 6.9 | 5.5 |
|  | 2 | 4.5 | 6.5 | 4.5 |
|  | 3 | 4.5 | 5 | 4 |
|  | 4 | 5 | 5.5 | 5 |
| **8** | 1 | 8 | 10 | 7.5 |
|  | 2 | 6.5 | 8.1 | 6 |
|  | 3 | 8 | 9 | 7.5 |
|  | 4 | 9.5 | 10 | 7 |
| **9** | 1 | 6 | 7.5 | 6 |
|  | 2 | 5.5 | 6.5 | 4 |
|  | 3 | 5 | 6.3 | 3.5 |
|  | 4 | 4.5 | 5.5 | 4.5 |
| **10** | 1 | 6.5 | 8 | 3.5 |
|  | 2 | 5.6 | 8.5 | 5 |
|  | 3 | 6 | 8 | 5 |
|  | 4 | 5.5 | 6.5 | 3.5 |

SUPPLEMENTARY TABLE A.3

| **Experiment 2** | |  |  |  |
| --- | --- | --- | --- | --- |
|  |  |  |  |  |
| **BASELINE** |  |  |  |  |
|  | MEP amplitudes | % post-PAS to pre-PAS | | |
|  |  | 0 min | 30 min | 60 min |
| Subject | pre-PAS baseline (µV) | % | % | % |
| 1 | 243 | 123 | 135 | 144 |
| 2 | 130 | 245 | 117 | 134 |
| 3 | 209 | 140 | 167 | 112 |
| 4 | 554 | 85 | 142 | 116 |
| 5 | 661 | 154 | 134 | 137 |
| 6 | 241 | 263 | 195 | 168 |
| 7 | 238 | 158 | 70 | 87 |
| 8 | 282 | 109 | 66 | 88 |
| 9 | 366 | 120 | 95 | 90 |
| 10 | 183 | 122 | 115 | 119 |
| Mean |  | 152 | 124 | 120 |
| **Mean – 100%** |  | **52** | **24** | **20** |
| STD |  | 58 | 40 | 27 |
| **STE** |  | **18** | **13** | **8** |
|  |  |  |  |  |
|  |  |  |  |  |
| **25% HIGHER** | |  |  |  |
|  | MEP amplitudes | % post-PAS to pre-PAS | | |
|  |  | 0 min | 30 min | 60 min |
| Subject | pre-PAS baseline (µV) | % | % | % |
| 1 | 234 | 173 | 56 | 28 |
| 2 | 126 | 103 | 141 | 126 |
| 3 | 291 | 124 | 113 | 92 |
| 4 | 743 | 137 | 143 | 153 |
| 5 | 340 | 224 | 148 | 80 |
| 6 | 117 | 242 | 250 | 315 |
| 7 | 344 | 337 | 121 | 57 |
| 8 | 481 | 62 | 77 | 131 |
| 9 | 211 | 130 | 90 | 71 |
| 10 | 162 | 266 | 136 | 153 |
| Mean |  | 180 | 127 | 121 |
| **Mean – 100%** |  | **80** | **27** | **21** |
| STD |  | 85 | 53 | 80 |
| **STE** |  | **27** | **17** | **25** |
|  |  |  |  |  |
|  | |  |  |  |
| **25% LOWER** | |  |  |  |
|  | MEP amplitudes | % post-PAS to pre-PAS | | |
|  |  | 0 min | 30 min | 60 min |
| Subject | pre-PAS baseline (µV) | % | % | % |
| 1 | 186 | 159 | 132 | 160 |
| 2 | 318 | 130 | 89 | 89 |
| 3 | 185 | 195 | 152 | 137 |
| 4 | 335 | 174 | 232 | 111 |
| 5 | 525 | 239 | 246 | 195 |
| 6 | 144 | 196 | 157 | 272 |
| 7 | 246 | 97 | 55 | 48 |
| 8 | 216 | 105 | 137 | 122 |
| 9 | 158 | 232 | 161 | 194 |
| 10 | 216 | 196 | 108 | 117 |
| Mean |  | 172 | 147 | 145 |
| **Mean – 100%** |  | **72** | **47** | **45** |
| STD |  | 49 | 59 | 64 |
| **STE** |  | **16** | **19** | **20** |
|  |  |  |  |  |
|  | |  |  |  |
| **50% LOWER** | |  |  |  |
|  | MEP amplitudes | % post-PAS to pre-PAS | | |
|  |  | 0 min | 30 min | 60 min |
| Subject | pre-PAS baseline (µV) | % | % | % |
| 1 | 203 | 223 | 195 | 142 |
| 2 | 271 | 132 | 137 | 144 |
| 3 | 165 | 233 | 178 | 287 |
| 4 | 623 | 80 | 118 | 100 |
| 5 | 494 | 163 | 126 | 74 |
| 6 | 86 | 143 | 237 | 244 |
| 7 | 203 | 137 | 148 | 87 |
| 8 | 236 | 81 | 94 | 130 |
| 9 | 160 | 140 | 151 | 158 |
| 10 | 212 | 176 | 125 | 148 |
| Mean |  | 151 | 151 | 151 |
| **Mean – 100%** |  | **51** | **51** | **51** |
| STD |  | 51 | 42 | 67 |
| **STE** |  | **16** | **13** | **21** |
